# Digitization of Dentate and Edentulous Maxillectomy and Mandibulectomy Defects with Three Different Intraoral Scanners: A Comparative In Vitro Study

**DOI:** 10.3390/jcm13226810

**Published:** 2024-11-13

**Authors:** Mariko Hattori, Sandra Stadler, Yuka I. Sumita, Benedikt C. Spies, Kirstin Vach, Ralf-Joachim Kohal, Noriyuki Wakabayashi

**Affiliations:** 1 Department of Advanced Prosthodontics, Graduate School of Medical and Dental Sciences, Institute of Science Tokyo, 1-5-45 Yushima, Bunkyo-ku, Tokyo 113-8549, Japan; sasamfp@tmd.ac.jp (M.H.); wakabayashi.rpro@tmd.ac.jp (N.W.); 2 Private Dental Clinic, 93133 Burglengenfeld, Germany; sandra.stadler@online.de; 3 Department of Partial and Complete Denture, School of Life Dentistry at Tokyo, The Nippon Dental University, 1-9-20 Fujimi, Chiyoda-ku, Tokyo 102-8158, Japan; 4 Department of Prosthetic Dentistry, Center for Dental Medicine, Medical Center-University of Freiburg, Faculty of Medicine, University of Freiburg, Hugstetterstr. 55, 79106 Freiburg, Germany; benedikt.spies@uniklinik-freiburg.de (B.C.S.); ralf.kohal@uniklinik-freiburg.de (R.-J.K.); 5 Institute for Medical Biometry and Statistics, Medical Center-University of Freiburg, Faculty of Medicine, University of Freiburg, Hugstetterstr. 55, 79106 Freiburg, Germany; kirstin.vach@uniklinik-freiburg.de

**Keywords:** digital impression, maxillofacial prosthetics, dental model, precision, trueness

## Abstract

**Objectives**: The objective of this study was to compare the trueness and precision of three intraoral scanners (IOSs) for the digitization of dentate and edentulous maxillectomy and mandibulectomy defects in artificial models. **Methods:** Four representative defect models—a dentate and an edentulous maxillectomy model and a dentate and an edentulous mandibulectomy model—were used for digital scanning. After a reference scan of each model, they were scanned with three IOSs: CEREC AC Omnicam, True Definition, and cara TRIOS 3. For comparison, five conventional impressions with a polysiloxane material were taken and digitized with a laboratory scanner. The obtained data were evaluated with three-dimensional (3D) inspection software and superimposed with the reference scan data by using a best-fit algorithm. The mean absolute 3D deviations of the IOS compared to the reference data (trueness) and when comparing the datasets within the IOS (precision) were analyzed. Linear mixed models and multiple pairwise comparisons were used for statistical analyses. **Results:** The overall comparison of the four evaluated procedures for data acquisition showed a significant difference in trueness (*p* < 0.0001) and precision (*p* < 0.0001). The average mean trueness of the IOSs ranged from 32.17 to 204.43 µm, compared to 32.07 to 64.85 µm for conventional impressions. Here, the conventional impression and cara TRIOS 3 performed the most precisely with no significant difference. CEREC AC Omnicam achieved the worst precision. **Conclusions:** Using a suitable intraoral scanner, defective jaws even without teeth could be captured in satisfying accuracy. This shows the possibility to use an intraoral scanner for maxillofacial defect patients and gives a vision of using digital technology in maxillofacial prosthetics.

## 1. Introduction

Computerized dentistry is one of the most promising emerging fields in dentistry, with many innovations and rapid developments as well as a steadily growing user base [1,2]. Introduced in the 1980s for the chairside fabrication of single-unit dental restorations, computer-aided design (CAD) and computer-aided manufacturing (CAM) techniques now cover nearly the full range of clinical applications to date [3]. In particular, the resolution and potential applications of digital impressions taken with intraoral scanners (IOS) are rapidly improving [4]. At first, scanning of only a single tooth was feasible and efficient, but now the digitization of full arches is possible. Today, digital impressions of implants made using a so-called scan body have been well described and scientifically evaluated [5]. Numerous research articles and clinical reports have focused on the application of IOS to the digitization of both teeth and oral implants [1,6,7,8]. In 2013, Patzelt et al. were the first to report that scanning of the edentulous jaw is feasible in a laboratory setting [9]. This finding prompted interest in the use of digital impressions in the field of maxillofacial prosthodontics. Digital recording of maxillofacial defects could avoid the risk of aspiration, swallowing, and impaction of impression material and might reduce the discomfort of patients in comparison with conventional impressions. Capturing intraoral defects by using an IOS is expected to be safer and more comfortable for both the patient and the dentist [10,11]. In 2017, Elbashti and colleagues showed that scanning of a maxillectomy model appears to be accurate and feasible [12]. They also compared the accuracy between different defect groups [13]. Zhang and colleagues showed that scanning of the jaw after maxillectomy was possible in vitro [14]. These studies have demonstrated the possibility of using IOS in patients who have undergone tumor resection. However, the use of a single IOS to digitize edentulous maxillary models was noted as a limitation of previous work. Also, all these previous studies had the limitation of focusing on only maxillectomy, not mandibulectomy. To overcome these limitations, the aim of this study was to compare the accuracy, defined as trueness and precision [15], among three IOSs and to compare the IOS results with the results of a mixed analog–digital workflow (conventional impressions with subsequent digitization of the impressions) for maxillectomy and mandibulectomy defects in both dentate and edentulous models. The null hypothesis of this study was as follows: There are no significant differences between conventional impressions and digital impressions obtained with different IOSs in terms of trueness and precision.

## 2. Materials and Methods

### 2.1. Defect Models

The maxillectomy and mandibulectomy models were prepared using four commercially available epoxy resin models:

1. Maxillary Model with Partial Dentition (E50-525);
2. Mandibular Model with Full Dentition (E50-520);
3. Edentulous Maxillary Model (G1-402 U);
4. Edentulous Mandibular Model (G1-402 L).

**Figure 1:**
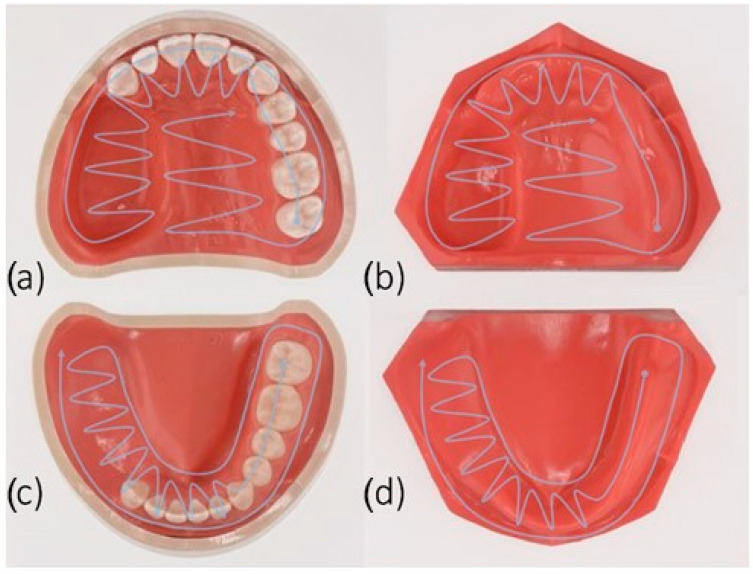
(**a**,**b**) Dentate and (**b**,**d**) edentulous reference models to be digitized with 4 different systems: (**a**) dentate maxillectomy model (MaxD), (**b**) edentulous maxillectomy model (MaxE), (**c**) dentate mandibulectomy model (ManD), (**d**) edentulous mandibulectomy model (ManE). Scanning strategies are shown as a gray arrow.

These are all Nissin Dental Products, Inc., Kyoto, Japan, as shown in Figure 1. The models were named as follows: dentate maxillectomy model (MaxD), dentate mandibulectomy model (ManD), edentulous maxillectomy model (MaxE), and edentulous mandibulectomy model (ManE). The dimensions of the upper jaw defect were about 33 mm (L) × 20 mm (W) × 25 mm (D), and those of the lower jaw defect were about 40 mm (L) × 20 mm (W) × 18 mm (D).

**Figure 2:**
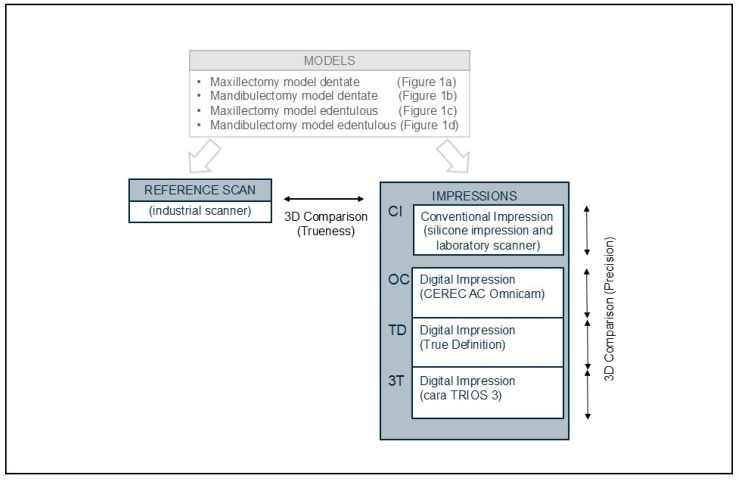
Data-acquisition process.

The process of data acquisition is shown in Figure 2. There were 5 scanners used in this study.

1. Industrial scanner (ATOS III Triple Scan 8 MP, GOM, Braunschweig, Germany);
2. Intraoral scanner named as OC (CEREC AC Omnicam, Sirona, Bensheim, Germany);
3. Intraoral scanner named as TD (True Definition, 3M ESPE, St. Paul, MN, USA);
4. Intraoral scanner named as 3T (cara TRIOS 3, Heraeus, Hanau, Germany);
5. Laboratory scanner (i/s/can, Organical CAD/CAM, Berlin, Germany).

The models were first digitized using an industrial scanner with a measurement error of 0.001 mm. Data acquired with the industrial scanner were used as a reference to calculate the trueness of the different systems evaluated (3 IOS and 1 conventional impression followed by a laboratory scan).

### 2.2. Intraoral Scanners (IOS)

For the scanning of the jaws, the three IOSs (OC, TD, and 3T) were used. To enable digitization with the TD system, the surfaces were slightly dusted with a titanium dioxide and zirconium oxide powder (3M ESPE Lava Powder, 3M ESPE) per the manufacturer’s instructions. For the OC and 3T systems, the powder was not used because it was not mentioned in the manufacturers’ instructions.

### 2.3. Execution of the Digital Scans

Scans were performed according to the manufacturers’ recommendations for full arch scans: The jaw defects were scanned in a spiral method as shown in Figure 1. All scans were taken in the same room excluding the influence of extraneous light and under the same temperature and humidity conditions (mean temperature 22 ± 1 °C; relative humidity 45 ± 5%). The scanning time for each system was recorded (n = 80) (Table 1). The hardware startup, software setting, and powder application was not included in the recorded time. Afterwards, the quality of the scans was confirmed on the monitor of the scanning device. Data obtained with the TD scanner were sent to the provider (3M ESPE) for further post-processing. Subsequently, the data were saved in Standard Triangulation/Tessellation Language (STL) format. The remaining scanners (OC, 3T) allowed the data to be directly exported into the STL format.

### 2.4. Conventional Impression with Subsequent Laboratory Scan

Conventional impressions were made with a two-phase, one-step technique using a hydrophilic vinyl polysiloxane impression material (Exafine Regular and Injection, GC Corporation, Tokyo, Japan). Individual trays were prepared using a self-curing acrylic resin (Ostron 100, GC). The border molding was conducted using an impression compound (Iso-functional, GC) to fill the gap between the margin of the tray and the model. A tray adhesive (GC) was applied to the inside and outside of the individual tray and left to dry for 1 min. The processing time was measured from the mixing of the impression material until the removal of the jaw model after the final setting of the material. The impression was stored at least 2 h to ensure elastic recovery and was poured with type III dental stone (Pico-crema soft, Picodent, Wipperfürth, Germany) following the manufacturer’s instructions. The casts were trimmed and dried for 24 h before scanning with the laboratory scanner. The optical scan data were saved directly as STL files.

### 2.5. Alignment and Measurement Procedure

The data files obtained were loaded into 3D inspection software (Geomagic Control X 2017, Geomagic, Morrisville, NC, USA). After the removal of artifacts, datasets were cropped proximal to the vestibule. As in previous studies evaluating general trueness, the datasets were superimposed on the reference jaw model by using a best-fit algorithm [9,14]. For general precision, the different datasets (n = 5) obtained from the same model were compared for each of the different systems.

### 2.6. Sample Size Calculation

With a sample size of n = 5 scans per device and an expected standard deviation of approximately 20 µm for the most relevant outcome (based on a comparable study in the literature by Patzelt et al. [9]), a mean difference of about 170 µm between the groups could be detected with a power of 93%. As a result of sample size calculation, each model (n = 4) was scanned 5 times with all workflows to be evaluated (n = 4), resulting in a total of 80 scans/datasets.

### 2.7. Statistical Analysis

Mean absolute 3D deviations were determined and statistically analyzed for precision and trueness. Every model was scanned 5 times and each scan was converted into graph structured data, reflecting only the average of the positive and negative deviations. The scans were modeled by a discrete set of points. At each measurement point, the deviation from the reference had a sign, positive or negative. To accurately evaluate the readings, cancellation was taken into account and both positive and negative mean variations were measured, thereby eliminating false optimum values.

For descriptive analysis, the mean, median, and standard deviation (SD) were computed. Furthermore, boxplots were used for graphical presentation of the data. Linear mixed models were fitted with random intercepts for each sample to evaluate device effects on “mean deviations” for trueness and precision. Furthermore, pairwise comparisons were made. The Scheffé method was used to correct for multiple comparisons. All calculations were performed using the statistical software STATA 14.2. The level of statistical significance was set at *p* < 0.05.

## 3. Results

The four models were digitized using the three IOSs and impressions of the models were taken in the conventional method group, finally resulting in 80 STL files to be evaluated (20 stone casts and 60 IOS datasets). The mean (SD) times for impressions are shown in Table 1. In some cases, the scanning was interrupted. Image stitching in areas without complex surface topography seemed to be complicated for the IOSs and the reference surface for alignment was lost. Because of the complexity of the surface topography, this happened more frequently when digitally scanning the edentulous models. However, all scans could be obtained with correct stitching of the data.

**Figure 3:**
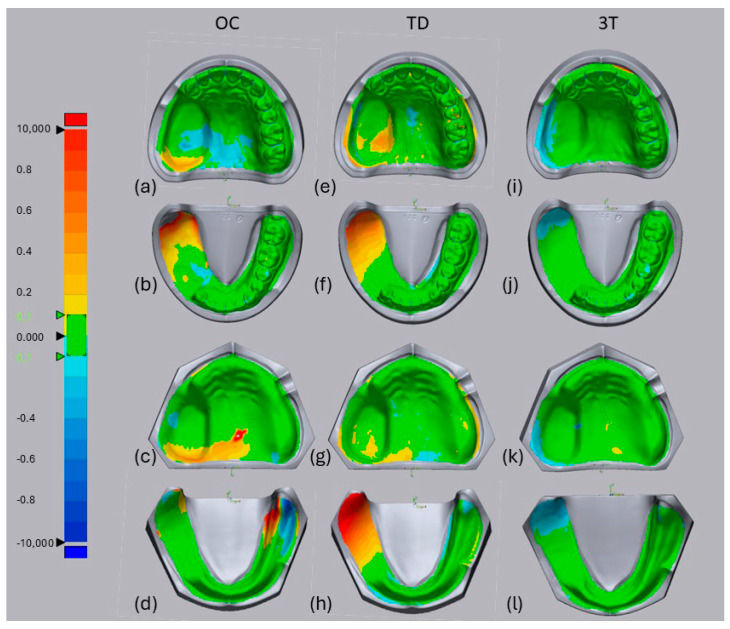
Representative datasets of an intraoral scanner visualized by Geomagic Control X software using OC, CEREC AC Omnicam (**a**–**d**), TD, true definition (**e**–**h**), 3T, cara TRIOS 3 (**i**–**l**) of dantate maxillectomy (**a**,**e**,**i**), dentate mandibulectomy (**b**,**f**,**j**), edentulous maxillecomy (**c**,**g**,**k**) and edentulous mandibulectomy (**d**,**h**,**l**) models. Color scale units: mm.

For the TD system, visual analysis of the superimposed data for the lower jaw (Figure 3f,h) revealed a positive misfit of the posterior and lateral defect walls (shown in red) compared with the reference scan. The edentulous non-defect sides and posterior defect walls were difficult for the OC system (Figure 3d), whereas the 3T system mostly showed a good fit (green) except for a few parts showing a slightly negative misfit at the posterior side of the defect compared with the reference model (Figure 3j,k, blue). Table 2 shows the mean three-dimensional deviation (standard deviation) in micrometers for trueness and precision. An overall comparison of the four different impression methods showed a significant difference in trueness (*p* < 0.0001) and precision (*p* < 0.0001). The 3T scanner was the most true (43.40 µm), followed by CI (48.38 µm). In terms of precision, both of these systems were likewise evaluated as the most precise (3T: 26.90 µm; CI: 27.03 µm). The OC and TD scanners were significantly less true and less precise. Multiple pairwise comparisons of trueness and precision among the systems are shown in Table 3. For the different IOSs, 3T achieved the highest trueness with significantly better values compared with OC (*p* = 0.005) and TD (*p* = 0.005). In comparison with CI, only 3T did not show a significant difference in trueness (*p* = 0.990), which means that 3T and CI performed similarly well. Comparing the precision of the four methods, only OC showed a significant difference compared with 3T (*p* = 0.000), TD (*p* = 0.000), and CI (*p* = 0.000). OC was the least precise among all of the IOSs and CI.

**Figure 4:**
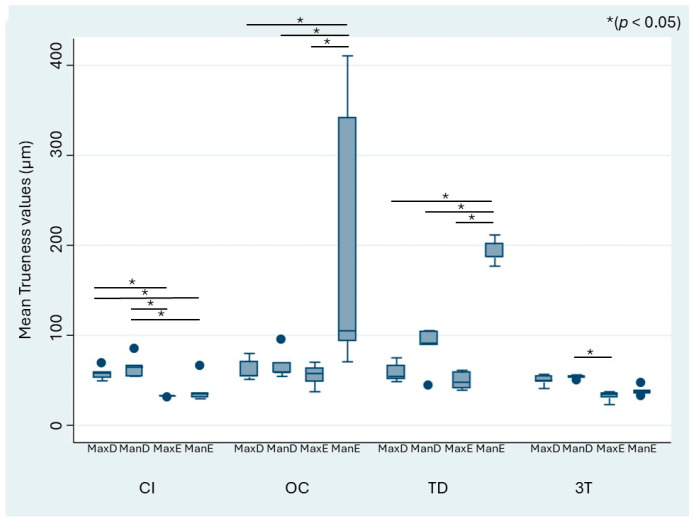
Mean absolute trueness values and statistically significant differences between the scanners (* *p* < 0.05). Circles represent outliers. MaxD, dentate maxillectomy model; ManD, dentate mandibulectomy model; MaxE, edentulous maxillectomy model; ManE, edentulous mandibulectomy model.

**Figure 5:**
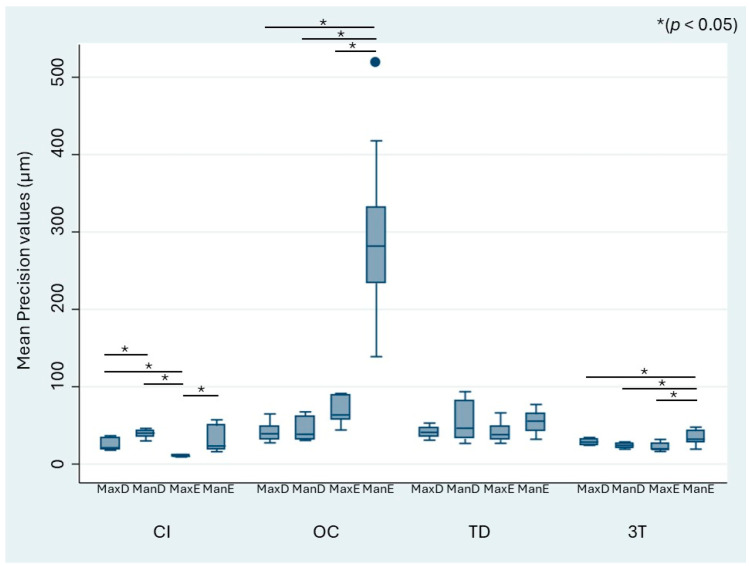
Mean absolute precision values and statistically significant differences between the scanners (* *p* < 0.05). Circles represent outliers. MaxD, dentate maxillectomy model; ManD, dentate mandibulectomy model; MaxE, edentulous maxillectomy model; ManE, edentulous mandibulectomy model.

When stratified by the different defect models, the mean trueness values of the IOS ranged from 32.17 to 204.43 µm, whereas those of CI ranged from 32.07 to 64.85 µm (Figure 4). The OC and TD scanners showed a significant difference in trueness for the digitization of the edentulous mandibulectomy model (ManE) when compared with the other models (MaxD, MaxE, ManD). OC and TD performed the worst in terms of trueness for ManE. For CI, the acquired datasets showed general heterogeneity. Figure 5 similarly shows the precision values stratified by the model and impression technique. The mean precision of the IOS systems ranged from 21.34 to 68.85 µm, whereas that of CI ranged from 10.49 to 39.62 µm. As observed for trueness, OC showed significantly higher differences in precision for the edentulous mandibulectomy jaw (ManE) data compared with the MaxD, MaxE, and ManD models.

## 4. Discussion

The null hypothesis of this study was rejected because there were significant differences between the conventional impressions and the digital impressions obtained with different IOSs. To the authors’ knowledge, this in vitro study is the first to digitize edentulous and dentate maxillectomy and mandibulectomy defect models with three different IOSs. The data obtained were compared with the data of conventional impressions digitized with a laboratory scanner. To create the reference dataset, an industrial scanner was chosen because of its high accuracy [16,17]. The IOS systems were selected because they are widely used in clinical practice and are commercially available internationally. Conventional impressions were taken by an experienced dentist (S.S.) using a conventional technique with a hydrophilic vinyl polysiloxane impression material in accordance with standard practice in dentistry [18]. The 3D inspection software used in this study (Geomagic Control X 2017) is widely used for superimposition and alignment of 3D comparisons in digital dentistry [9,10,11]. Although some researchers have used the overall mean 3D deviation, maximum and minimum mean values were used in the present investigation as in the study by Patzelt and co-workers [9]. In their study, the procedure was stated to result in increased preciseness of the outcome. Despite the presence of a large jaw defect, all scans were successfully performed in a similar time compared with the scanning of dentate non-defect models [9,10,19,20], implicating the feasibility to use IOSs for the digital scanning of resected jaws. A multiple pairwise comparison of all procedures for surface data acquisition showed that the deviations in the trueness of OC and TD were significantly higher than those of CI and 3T. There was no significant difference between 3T and CI. In terms of precision, only the OC values were significantly worse compared with the other procedures. There were no significant differences between CI, TD, and 3T. The results of the present study are comparable with previous studies evaluating trueness and precision when digitally recording different dentate jaw models with IOSs [2,9,11,21].

When scanning the edentulous mandibulectomy model, the OC values had a large deviation range up to 400 µm, showing significant differences compared with all the other models in both trueness and precision. TD showed a strong deviation in trueness for the edentulous mandibulectomy model but small deviations in precision. The small deviations in precision suggest that the error might be system-specific, indicating that the TD has the same tendency of deviation at every scan. A possible explanation of the higher deviations in trueness could be a lack of characteristic landmarks in the edentulous lower jaw model. The maxillectomy defect model used had a framed shape while the mandibulectomy defect model appeared to have a flatter surface without any visible borders. Visual analysis demonstrated that all of the IOSs used showed the maximum deviation at different parts of the model. Therefore, the location of failure might be inherent to the system and cannot be clearly linked to the morphology of the evaluated models. Difficulties with posterior defect walls could be caused by the structural complexity of the defect.

Compared with the data of digitized edentulous models without defects reported by Patzelt et al. [9], the three IOSs used in the present study showed similar ranges of trueness and precision for the mandible. For the maxilla models, the present data showed smaller values of trueness and precision, which can probably be explained by the development of the scanners. In the present study, the trueness and precision for the edentulous maxillectomy jaw model showed decreased deviation compared with the findings of Elbashti et al. [11]. It is assumed that this might be due to the use of human models in the previous work, which showed an increased variation in morphology. In comparison to the findings of Imburgia et al. [5], who found that trueness was better for partially edentulous models than for fully edentulous models, only the OC and TD scanner showed a significantly better trueness for the dentate models compared with the edentulous mandibulectomy model in this study. Finally, the deviations for the present dentate jaws were slightly higher than those in previous studies on full arch dentate scans [2,14]. The presence of defects might be an explanation for this.

The results of this study suggest that intraoral scanners can be effectively used in clinical settings to digitize both dentate and edentulous maxillofacial defects. This advancement could significantly benefit clinical practitioners by providing a safer and more comfortable alternative to conventional impression techniques. The integration of IOS into clinical workflows may enhance the precision of prosthetic fabrication, reduce chair time, and improve patient satisfaction. As digital technology continues to evolve, its application in maxillofacial prosthetics could lead to more efficient and accurate treatment planning and execution.

The limitations of this study include that its findings are from an in vitro setting that was controlled to avoid interfering effects. In this study, the outlines of the impression were trimmed uniformly proximal to the vestibule; however, in vivo, it is difficult to determine where to trim, and accuracy is affected by the position of the border. If digital impressions were taken in vivo, the accuracy of the movable mucosa would be reduced; therefore, the results from an in vivo setting are expected to be less accurate compared with the present results. Moreover, the models used are special-case scenarios and cannot be considered generally representative of complex clinical defects [11,22]. Furthermore, the models had color and reflection values different from those of real teeth and soft and hard tissues in the oral cavity. In addition, this study did not simulate oral conditions such as saliva or mobile tissues. Factors such as patient movement and compliance were also not simulated but can be expected to make the use of IOSs more challenging in the clinical setting. Furthermore, many patients have restricted mouth opening after maxillectomy or mandibulectomy. In such patients, the head size of the IOS will be a crucial factor in whether a digital impression is possible.

Despite the limitations above, this study has demonstrated the potential feasibility of the chairside use of IOSs for jaw defects. The use of IOSs in maxillectomy or mandibulectomy patients warrants further investigation in the clinical setting.

Given the observed variations in accuracy among different intraoral scanners, it is crucial to establish standardized protocols to ensure consistent outputs. The development of industry-wide guidelines for the calibration and maintenance of IOS devices is recommended. Regular software updates and training for practitioners on optimal scanning techniques could further enhance accuracy. Additionally, collaboration between manufacturers to establish common standards for data output and processing could help to mitigate discrepancies and improve the reliability of digital impressions in maxillofacial prosthetics.

## 5. Conclusions

Despite the limitations of this model study, the results suggest that both dentate and edentulous jaws with defects can be captured with satisfactory accuracy using an IOS. This study demonstrates the potential feasibility of using an IOS in maxillofacial defect patients and hints at a future where digital technology is practically applied in maxillofacial prosthetics.

## Figures and Tables

**Table 1:** Mean values and standard deviation for the recorded times (min:s).

Impression System	MaxD	ManD	MaxE	ManE
CI	3:43 (0.09)	3:17 (0.05)	3:52 (0:06)	3:36 (0:11)
OC	4:38 (0:31)	4:57 (0:22)	4:35 (0:38)	6:13 (0:27)
TD	4:42 (0:14)	4:35 (0:07)	4:55 (0:10)	4:42 (0.04)
3T	3.25 (0:07)	2:49 (0:12)	4:00 (0:15)	4:15 (0:10)

CI, conventional impression; OC, CEREC AC Omnicam; TD, true definition; 3T, cara TRIOS 3.

**Table 2:** Mean values and standard deviation for the trueness and precision of the different systems.

Impressions	Mean (SD) Trueness, µm	Mean (SD) Precision, µm
CI	48.38 (16.55)	27.03 (14.21)
OC	97.20 (97.52)	111.90 (119.84)
TD	96.97 (60.21)	48.38 (17.69)
3T	43.30 (9.99)	26.90 (7.33)

CI, conventional impression; OC, CEREC AC Omnicam; TD, true definition; 3T, cara TRIOS 3.

**Table 3:** Multiple pairwise comparisons of the trueness and precision of the different systems (Scheffé-adjusted).

Impressions	*p* Values for Trueness	*p* Values for Precision
OC vs. CI	0.015 *	<0.0001 *
TD vs. CI	0.015 *	0.380
3T vs. CI	0.990	0.983
TD vs. OC	1.000	<0.0001 *
3T vs. OC	0.005 *	<0.0001 *
3T vs. TD	0.005 *	0.179

CI, conventional impression; OC, CEREC AC Omnicam; TD, true definition; 3T, cara TRIOS 3. * *p* values of < 0.05 were considered statistically significant.

## Data Availability

Data are available from Mariko Hattori [sasamfp@tmd.ac.jp] for researchers who meet the criteria for access to confidential data.

## References

[1] Zimmermann M., Mehl A., Mörmann W.H., Reich S. (2015). Intraoral scanning systems—A current overview. Int. J. Comput. Dent..

[2] Ender A., Attin T., Mehl A. (2016). In vivo precision of conventional and digital methods of obtaining complete-arch dental impressions. J. Prosthet. Dent..

[3] Sannino G., Germano F., Arcuri L., Bigelli E., Arcuri C., Barlattani A. (2015). CEREC CAD/CAM Chairside System. Oral Implantol..

[4] Rutkūnas V., Gečiauskaitė A., Jegelevičius D., Vaitiekūnas M. (2017). Accuracy of digital implant impressions with intraoral scanners. A systematic review. Eur. J. Oral Implantol..

[5] Imburgia M., Logozzo S., Hauschild U., Veronesi G., Mangano C., Mangano F.G. (2017). Accuracy of four intraoral scanners in oral implantology: A comparative in vitro study. BMC Oral Health.

[6] Patzelt S.B., Emmanouilidi A., Stampf S., Strub J.R., Att W. (2014). Accuracy of full-arch scans using intraoral scanners. Clin. Oral Investig..

[7] Chochlidakis K.M., Papaspyridakos P., Geminiani A., Chen C.J., Feng I.J., Ercoli C. (2016). Digital versus conventional impressions for fixed prosthodontics: A systematic review and meta-analysis. J. Prosthet. Dent..

[8] Papaspyridakos P., Gallucci G.O., Chen C.J., Hanssen S., Naert I., Vandenberghe B. (2016). Digital versus conventional implant impressions for edentulous patients: Accuracy outcomes. Clin. Oral Implant. Res..

[9] Patzelt S.B., Vonau S., Stampf S., Att W. (2013). Assessing the feasibility and accuracy of digitizing edentulous jaws. J. Am. Dent. Assoc..

[10] Patzelt S.B., Lamprinos C., Stampf S., Att W. (2014). The time efficiency of intraoral scanners: An in vitro comparative study. J. Am. Dent. Assoc..

[11] Elbashti M.E., Hattori M., Patzelt S.B., Schulze D., Sumita Y.I., Taniguchi H. (2017). Feasibility and Accuracy of Digitizing Edentulous Maxillectomy Defects: A Comparative Study. Int. J. Prosthodont..

[12] Elbashti M.E., Hattori M., Patzelt S.B., Aswehlee A.M., Sumita Y., Taniguchi H. (2019). Precision and Trueness of Computerized Optical Impressions in Maxillectomy Defects: An In Vitro 3D Comparison. Int. J. Prosthodont..

[13] Zhang M., Hattori M., Elbashti M.E., Sumita Y.I. (2020). Feasibility of Intraoral Scanning for Data Acquisition of Maxillectomy Defects. Int. J. Prosthodont..

[14] Malik J., Rodriguez J., Weisbloom M., Petridis H. (2018). Comparison of Accuracy Between a Conventional and Two Digital Intraoral Impression Techniques. Int. J. Prosthodont..

[15] (2023). Accuracy (Trueness and Precision) of Measurement Methods and Results, Part 1: General Principles and Definitions.

[16] Matta R.E., Bergauer B., Adler W., Wichmann M., Nickenig H.J. (2017). The impact of the fabrication method on the three-dimensional accuracy of an implant surgery template. J. Craniomaxillofac. Surg..

[17] Nedelcu R., Olsson P., Nyström I., Thor A. (2018). Finish line distinctness and accuracy in 7 intraoral scanners versus conventional impression: An in vitro descriptive comparison. BMC Oral Health.

[18] Donovan T.E., Chee W.W. (2004). A review of contemporary impression materials and techniques. Dent. Clin. N. Am..

[19] Besl P.J., McKay N.D. (1992). A method for registration of 3-D shapes. IEEE Trans. Pattern Anal. Mach. Intell..

[20] Yuzbasioglu E., Kurt H., Turunc R., Bilir H. (2014). Comparison of digital and conventional impression techniques: Evaluation of patients’ perception, treatment comfort, effectiveness and clinical outcomes. BMC Oral Health.

[21] Nedelcu R., Olsson P., Nyström I., Rydén J., Thor A. (2018). Accuracy and precision of 3 intraoral scanners and accuracy of conventional impressions: A novel in vivo analysis method. J. Dent..

[22] Beumer J., Beumer J., Marunick M.T., Esposito S.J. (2011). Rehabilitation of maxillary defects. Maxillofacial Rehabilitation: Prosthodontic and Surgical Management of Cancer-Related, Acquired, and Congenital Defects of the Head and Neck.

